# Assessing the value of orphan drugs using conventional cost-effectiveness analysis: Is it fit for purpose?

**DOI:** 10.1186/s13023-022-02283-z

**Published:** 2022-04-05

**Authors:** Maarten J. Postma, Declan Noone, Mark H. Rozenbaum, John A. Carter, Marc F. Botteman, Elisabeth Fenwick, Louis P. Garrison

**Affiliations:** 1 Department of Health Sciences, University Medical Center, University of Groningen, Groningen, The Netherlands; 2 Department of Economics, Econometrics and Finance, Faculty of Economics and Business, University of Groningen, Groningen, The Netherlands; 3 European Haemophilia Consortium, Brussels, Belgium; 4 Pfizer, Inc., Rotterdam, The Netherlands; 5 OPEN Health – Evidence & Access, Bethesda, USA; 6 OPEN Health – Evidence & Access, Oxford, UK; 7 Comparative Health Outcomes, Policy, and Economics Institute, University of Washington, Seattle, USA

**Keywords:** Orphan drug policy, Pharmaceutical policy, Rare diseases, Value-based pricing, Health equity, Value of life

## Abstract

Conventional cost-effectiveness analysis—i.e., assessing pharmaceuticals through a cost per quality-adjusted life year (QALY) framework—originated from a societal commitment to maximize population health given limited resources. This "extra-welfarist" approach has produced pricing and reimbursement systems that are not well- aligned with the unique considerations of orphan drugs. This framework has been slow to evolve along with our increased understanding of the impact of rare diseases, which in turn has complicated the assessment of orphan drugs meant to treat rare diseases. Herein, we (i) discuss the limitations of conventional cost-effectiveness analysis as applied to assessing access to, as well as the pricing and reimbursement of, orphan drugs, (ii) critically appraise alternative and supplemental approaches, and (iii) offer insights on plausible steps forward.

## Background

**Fig. 1:**
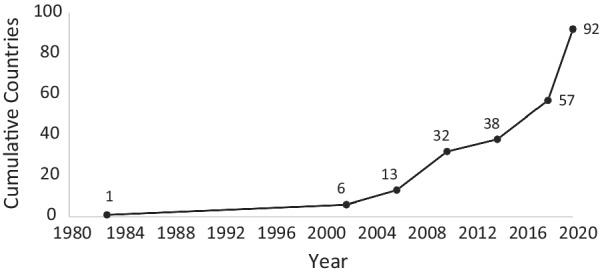
Cumulative number of countries with orphan drug policies

First defined in the United States by the 1983 Orphan Drug Act, orphan drugs are products that could address an unmet clinical need but have low investment potential, primarily due to the small size of the affected population [1]. Approximately 50% of countries (Fig. 1) have enacted policies that support orphan drug research and development through strategies that include guaranteed market exclusivity, tax credits, and accelerated approval. These incentives are meant to encourage development of products to address the health needs of the 4% of the global population with a rare disease [2–5].

These orphan drug policies are controversial. On the one hand, some argue that pro-orphan drug policies address important unmet needs among persons who might otherwise experience barriers to timely and effective care and who tend to be younger and have more severe health issues [2, 5, 6]. They cite low associated per-capita spending [7] and the small share of pharmaceutical budgets attributable to orphan drugs [8] as evidence that the budgetary impact of orphan drugs is mitigated by the low prevalence of their indications. On the other hand, society bears the cost of subsidizing orphan drug development even though the acquisition costs of orphan drugs have outpaced increases in drug spending for common indications [3, 9, 10]. Further still, these policies are susceptible to manipulation, for example via “partial” orphan strategies whereby a drug is first approved for an orphan indication then for a common one but without an associated adjustment to the price [9, 11–13]. Both arguments are reasonable, but neither is complete without also considering the value that orphan drugs generate for patients with rare diseases and the broader society. Despite their mostly low overall budget impact, orphan drugs can be costly, but are they worth it?

The process for determining economic value, the extent of a new drug’s availability, and the appropriate level of reimbursement is often addressed by conducting a cost-effectiveness analysis (CEA) [14] to determine if it is worth paying the additional cost associated with a new drug given the additional benefit it conveys. In conventional CEA, this metric is represented in terms of the incremental cost per quality-adjusted life-year (QALY) gained (known otherwise as the incremental cost-effectiveness ratio [ICER]) and, to reach a decision, is compared to a pre-defined or revealed willingness-to-pay threshold. This decades-old approach is embedded within the traditional drug research and development paradigm. It has focused on the most prevalent diseases, but for several reasons, evaluating orphan drugs in this way is inadequate and—as is noted subsequently in this commentary—other options are needed [15].

Medical science has recently progressed to the point where we can develop treatments for our most rare diseases. However, conventional CEA methods have not kept pace in terms of considering the unique ways that rare diseases affect society [16], nor how society prioritizes the needs of persons with rare diseases [17]. For example, some rare diseases disproportionately affect: patients by causing severe and lifelong disability and reduced quality of life [18]; caregivers by imposing a burden on their quality of life and ability to work, etc. [18]; and society via high long-term care costs and requirements for specialist expertise [6, 19]. Fundamentally, this is a debate between “improving total health—the objective underpinning conventional CEA—and equity objectives, such as reducing social inequality in health or prioritizing the severely ill” [20]. Herein, we (i) discuss the limitations of conventional cost- effectiveness analysis as applied to assessing access to as well as the pricing, and reimbursement, of orphan drugs, (ii) critically appraise alternative and supplemental approaches, and (iii) offer insights on plausible steps forward.

Note that a rare disease needn’t necessarily be a severe disease. However, rare diseases are more often associated with shortened lifespans and moderate-severe symptoms compared to non-rare diseases, and so here we are referring to rare, serious diseases when using the term rare unless noted otherwise.

## Practical and theoretical issues

Many issues complicate the evaluation of orphan drugs using conventional CEA. We have organized these into three categories: (1) conflict of basic principles—how the nature of orphan drugs conflicts with the basic motivating principles for conventional CEA as applied to new medicines; (2) the complex nature and limited scope of the QALY metric—how issues in measuring health benefits for orphan drugs using QALYs play out; and (3) elevated uncertainty—how the valuation of orphan drugs exacerbates uncertainty within the conventional cost-effectiveness framework.

### Conflict of basic principles

Perhaps the most critical issue that complicates applying conventional CEA to orphan drugs is a conflict between the nature of orphan drugs and the motivations for using conventional CEA in the first place. This issue has been explored in the literature, where it is often discussed in terms of the intersection of ethics and economics and conflicts between ideals. One such conflict involves horizontal versus vertical equity. The former emphasizes equal treatment of equals (i.e., a utilitarian approach)—for example, applying the same cost per QALY threshold for all diseases [21]. The latter emphasizes unequal (but equitable) treatment of the unequals and would describe a system that considers the rarity of disease in its valuation of new drugs [22, 23]. Another conflict is between utilitarianism versus non- abandonment, or in other words and as used within the context of health economics specifically, maximized good for all versus favoritism toward those in dire need [24, 25]. Lastly, there is "welfarism" (the idea that the individual knows best what is best for their own well-being, which is broader than just health, interest) versus "extra-welfarism" (the idea that democratically-agreed principles can place limits on individual freedom in pursuit of other goals such as equity) [26, 27]. Under welfarism, the emphasis is on individuals maximizing their well-being. Under extra-welfarism, social welfare involves factoring in considerations of equity of access, outcomes, and well-being.

These debates can be distilled into a question that that is not new [28, 29]: “What values do the public want their healthcare systems to use in evaluating technologies?”

Notably, a guiding principle of conventional cost-effectiveness analysis, e.g., as applied in the UK, is that regulators and policymakers have an implicit mandate from the public to use resources to maximize health for the whole population. At the same time, the general public has demonstrated morally-based preferences for reducing inequalities in health outcomes [6, 30, 31] (though not universally [32]), which deviates somewhat from the principles that underlie conventional cost-effectiveness analysis because it is at the expense of opportunity cost and maximized distributed utility.

With finite resources, the goal of rational resource allocation is to generate the greatest good (or maximized utility for society) [33, 34]. Expressing a product’s value in terms of incremental cost per QALY gained has been—but need not necessarily be—the manifestation of this utility principle [25, 35] as it relates to coverage and reimbursement decisions. Those supporting a purely utilitarian perspective contend that orphan drugs should be evaluated by the same criteria as used for treatments for more common diseases (e.g., 30,000 GBP per QALY in the United Kingdom) because to do otherwise would violate the mandate to maximize utility for the population as a whole [35]. Under these conditions, orphan drugs may be less likely to be cost-effective, particularly when a novel, costly orphan drug is being compared to a lower-priced standard of care. Opposed to this view are those ascribing to a Rawlsian principle of “maximin” by which those who are worst off in terms of health (i.e., those with orphan conditions with limited treatment options) should be prioritized in terms of access to resources. As a result, higher cost per QALY for orphan drugs may be acceptable if this improves the health of the worst off in society, and utility is therefore not necessarily maximized at the population level. Likewise, other may favor the ‘rule-of-rescue: the imperative to help identifiable persons at immediate risk of harm [36, 37], which can effectively maximize utility at the individual level but not at the population level [2, 15, 38].

Indeed, preference for this ideal over pure utilitarianism (in the way that it is defined by health economists) has been routinely demonstrated in preference research [39]. Many would agree that persons with severe rare diseases are either in greater need than the general public or, at least, should not have societal benefits curtailed for them simply because of the low prevalence of their disease [3, 40, 41]. Orphan drugs are more likely to be considered cost-effective (and potentially reimbursed) if the maximin or rule of rescue approaches were adopted. Some even argue that ‘rule of rescue’ is justified from a utilitarian perspective because it reinforces one’s view of living in a compassionate society [36]. Rule of rescue applies to severe or life-threatening disease conditions, but not necessarily to non-severe orphan diseases. Similarly, recent work using a welfarist approach suggests that utility-maximizing individuals would be willing to pay a higher risk premium for rare, health catastrophic conditions [38].

### Problematic nature of the QALY

Though many health economists contend that the QALY is a valid--or at least useful--metric, issues with its application in CEA have been well- documented [29, 42–44]. Briefly, three issues predominate in criticism of the QALY, as it is currently employed in conventional CEA.

First, the QALY, particularly when measured with generic instruments [45], may not fully capture the benefits and harms of a treatment, health state and so forth, because the domains covered by the measure may not adequately capture the context of the disease or treatment impact. For example, it was recently observed that the EQ-5D has some usefulness in measuring quality of life in persons with Duchenne Muscular Dystrophy, but that it lacks the precision of disease-specific instruments [46]. If there is a lack of differentiation among health states in terms of the associated utilities, then there will be a commensurate lack of treatment benefit.

Second, there exists a "disability paradox" whereby an affected population judges their health more positively than does the general population [47]. Where pharmacoeconomic analyses characterize health states based on utility values derived from patients’ perceptions, this paradox leads to an underestimate of the disease burden and therefore underestimates the value of an effective treatment. An economic analysis based on patient-derived utilities would then under-value the effective treatment relative to the general public’s preference.

Third, disregarding the established principle of diminishing returns [48], conventional CEA assumes that all QALYs are equal, which is particularly problematic for orphan drugs because of how severe their given clinical indications are. Consider for example two hypothetical diseases: hypothetical disease A (low severity, high prevalence) and hypothetical disease B (high severity, low prevalence). Hypothetical disease A has a baseline utility of 0.7, which improves to 1.0 after treatment with drug A. Hypothetical disease B has a baseline utility of 0.3, which improves to 0.6 after treatment with drug B (an orphan). Conventional CEA, looking at the absolute difference, would consider the effects of these two treatments equivalent even though most in society would consider the improvement in hypothetical disease B to be more important as it increases the utility by 100% [15] (i.e., consistent with the maximin principle previously mentioned). In other words, conventional CEA would consider QALY improvements of the same magnitude (e.g., + 0.3) to be equally valuable, disregarding that this improvement for someone with a more severe (e.g., rare, severe) disease starting at a lower utility might have a greater impact and therefore be more valuable.

Further, because disease A is more prevalent than disease B an efficient system, with finite resources, might then favor treatment A [15].

The above example assumes that consumers are risk-neutral when in fact the opposite has been demonstrated [49, 50]. In reality, consumers are affected by risk aversion such that the application of conventional CEA (which contends that “a QALY is a QALY is a QALY” [51]) can effectively undervalue treatment of a severe rare disease by a considerable margin because the conventional approach does not fully consider the benefits of averting the catastrophic consequences of a rare disease through effective treatment [49, 52]. In other words, the application of the QALY via CEA systematically and predictably biases the analysis against orphan drugs [53].

### Uncertainty

A key issue affecting the suitability of conventional CEA for orphan drugs is uncertainty regarding efficacy and safety at the time the products are introduced. Often arising from small trial size, a lack of randomization or comparator, and the need to use surrogate efficacy measures [54], uncertainty about the durability of long-term benefits (which is magnified for rare diseases where longitudinal data are often insufficient) imbues conventional CEA—which is driven by comparative clinical effectiveness—with even greater certainty [55, 56]. Alternatively, some orphan drugs are curative and so confer a ‘value of knowing’ that reduces uncertainty about the treatment response; although, it also adds a new element of uncertainty with respect to whether the ‘cure’ is maintained. Nevertheless, this reduced uncertainty should be considered a measurable benefit [15].

Other elements of uncertainty unique to, or at least exacerbated for, rare diseases include financial risks to payers caused by imprecise knowledge about the size of the rare disease population and the further impact of this on their ability to forecast future expenditures across orphan drugs and rare diseases [6, 57–59].

## Alternative and supplemental approaches

One needs only to observe the number of alternative and supplemental valuation, pricing, and reimbursement approaches (e.g., NICE HST and CDF, ad hoc adjustments in Sweden, etc.) to recognize the practical challenges that health systems face in incorporating rare disease products [60]. Owing to persistent debate over the suitability of conventional CEA for evaluating orphan drugs, [29, 61] several alternative approaches have been proposed.

Diseases treated by orphan drugs may affect society to a greater degree compared to non-rare diseases when making the comparison on a per-patient basis (i.e., when not accounting for prevalence) because these diseases tend to be more severe and to affect younger persons [6]. Thus, one way to more accurately and precisely apply CEA to orphan drugs might be to increase the scope of what is considered as a contributor to the treatment risk/benefit profile, while being mindful of double-counting [62]. This idea of expanding the valuation context has produced several alternative approaches that consider the presence and interaction of novel concepts such as both financial and health risk protection as well as the "value of hope" and "real option value." [15].

One such approach is multicriteria decision analysis (MCDA) which has been applied to healthcare decision-making since the late 1980s [63–65]. Its key advantage is a capacity to flexibly consider and integrate the complexity of these decision problems, from different stakeholder perspectives, in a more manageable context rather than an approach to those perspectives that addresses them individually or without a pre-specified construct [17, 65, 66]. Though regarded as one of the more feasible alternatives to conventional cost-effectiveness, MCDA is inherently limited by difficulties of collecting, organizing, and correctly interpreting the disparate information required to populate its comprehensive stakeholder perspective in a consistent manner that supports comparisons across diseases [67]. Though prespecified MCDA frameworks have been promulgated and applied (e.g., EVIDEM [68]), these dynamic frameworks—combined with the amount of information needed to populate them—are difficult to replicate across products and indications [69]—and particularly in the context of new patent-protected medicines.

There have been studies that extend the scope of CEA to consider sources of harm and benefit that seem intuitive yet are routinely excluded from conventional CEA. Broadly speaking, the "value flower" constructed by Lakdawala et al. [38] presupposes that not all relevant elements of value are included in conventional CEA. And it seeks to expand the valuation context by supplementing QALYs and direct medical costs with other elements of value, such as productivity loss, scientific spillovers (e.g., investment in a given technology might increase the probability or rate of other advancements), disease severity (e.g., the impact of the disease on an individual’s daily functioning), and the value of hope.

A novel supplement (i.e., an adjustment not requiring wholesale change) to the conventional framework would be to incorporate fiscal and insurance values. For example, Connolly et al. 2019 assessed the pharmacoeconomic value of treating hereditary transthyretin amyloidosis from a societal perspective in the Netherlands [70]. Health-related (i.e., direct medical) costs aside, this analysis is unique in its consideration of the public budgetary costs such as disability payments, pensions, taxes, and earnings [71]. Elsewhere, conventional CEA is limited by assuming that the benefits of a medical technology are only gained by the persons being treated, when in fact, these benefits are also accrued by those who are not (or not yet) sick through two forms of risk protection [38]. Physical risk protection pertains to reduced fear of a disease (e.g., Alzheimer’s or COVID-19) that is produced by treatments that make the “illness less unpleasant”. Financial risk protection is value created by covering the costs of treatment through a public or private insurance system. For example, we buy automobile insurance not to mitigate the risk of an accident but to reduce our financial exposure if an accident happens. A new technology that reduces harm during an accident (e.g., airbags) reduces the risk of catastrophic injury and therefore reduces our risk of having to pay financially catastrophic medical bills. These two elements—physical and financial risk protection—combine to form "insurance value" [38], which can be quantified in an economic analysis [72].

In addition, several recent analyses have expanded the cost-effectiveness valuation context by addressing or correcting for a definition of equity that more accurately reflects the definition employed by the public. Fundamentally, these analyses [20, 73–75] treat the question of equity not from the perspective of prevalence but instead as a matter of equal access (in timing and magnitude) to healthcare, regardless of prevalence. Additionally, this technique of "extended cost-effectiveness analysis" incorporates an analysis of financial risk protection, in particular to protect against risks of catastrophic expenditures, that indeed rare diseases and orphan drugs may pose if the reimbursement fails [73]. Separately, the concept of “fair-innings” has been proposed, which somewhat benefits persons with rare diseases as it prioritizes extending life and QALYs for younger persons who would not otherwise live for the duration of life considered to be acceptable.

Noting that conventional CEA can be biased against the more severely, or terminally, ill by not considering the impact of diminishing returns on QALY improvements, Lakdawalla et al. [52] advocate for the generalized risk- adjusted cost-effectiveness (GRACE) approach by which quality of life returns diminish in the same way that non- health consumption gains do. For example, $10,000 given to a person who earns $500,000 per year does not have the same impact as $10,000 given to a person who earns $50,000 per year. This approach then corrects the discriminatory impact of conventional CEA against more severe illnesses.

Lastly, there are many instances where researchers advocate for increasing the cost-per-QALY threshold (for example, this has been implemented in the United Kingdom and the Netherlands in specific cases) such that it is aligned with societal preferences [6, 76, 77] that often emphasize the need to consider rarity, severity, patient age, and unmet needs when deriving the ICER threshold.

## Conclusions

Conventional CEA was developed when the prevalence, heterogeneity, and outsized economic impact of rare diseases were poorly understood and there were no effective treatments. This has produced a pharmacoeconomic valuation context that continues to emphasize a particular extra-welfarist utilitarian approach to societal healthcare resource allocation that favors treatments for more common (and often less devastating) diseases. Policies for administering CEA at the national payer level have not kept pace with technological and medical advances that are yielding effective orphan products. The resulting conflict undermines appropriate valuation of orphan drugs [76, 78], and one might argue also that the limitations of conventional CEA discussed here extend to the valuation of all medical products to some degree. However, the content and recommendations in this report pertain specifically to the application of conventional CEA to rare, severe diseases.

The shortcomings of conventional CEA with respect to orphan drugs centers primarily on three points: a conflict between equity and equality, limitations of the conventional application of the QALY, and how to deal with uncertainty. We have briefly summarized here several alternative approaches that have been developed or otherwise applied to address these issues and have found that these alternative approaches are primarily characterized by an emphasis on expanding the valuation framework to deal with considerations affecting value such as uncertainty and equity. MCDA and "augmented" cost-effectiveness analysis (per the "ISPOR value flower") are the most sensible and objective alternatives to conventional CEA. Arguably, we have seen some countries (e.g., UK and the Netherlands) implement adjustments to their conventional approach that addresses these concerns, such as a variable ICER threshold that considers disease severity [79]. Still, one could argue that no single alternative CEA approach fully considers non-conventional but meaningful benefits such as financial and health risk protection and altruistic value related to equity. Moreover, some of the arguments and proposed alternatives described herein represent somewhat extreme scenarios, while the middle-ground (e.g., value flower) should not be overlooked.

The question of how to value orphan drugs is not just technical—but also moral—because society’s needs are not fully addressed without addressing the suffering of the worst-off. It is, however, difficult to reconcile this moral disposition with conventional CEA. Society might instead benefit from an alternative or augmented cost- effectiveness framework that is flexible and varied in a more comprehensive, objective, and reproducible way.

## Abbreviations

CDF: Cancer Drugs Fund
CEA: Cost-effectiveness analysis
EMA: European Medicines Agency
GRACE: Generalized risk-adjusted cost-effectiveness
HST: Highly specialized technologies (guidance)
ICER: Incremental cost-effectiveness ratio
ICUR: Incremental cost-utility ratio
MCDA: Multi-criteria decision analysis
NHS: National Health Service (United Kingdom)
NICE: National Institute for Health and Care Excellence (United Kingdom)
QALY: Quality-adjusted life-year
